# Prevalence and sensitization of pollen–food allergy syndrome among adolescents in Tokyo

**DOI:** 10.1016/j.jacig.2025.100561

**Published:** 2025-08-28

**Authors:** Tomoyuki Kiguchi, Tomoki Yaguchi, Tatsuki Fukuie, Yukihiro Ohya, Kiwako Yamamoto-Hanada

**Affiliations:** a Allergy Center, National Center for Child Health and Development, Tokyo, Japan; b Medical Support Center for the Japan Environment and Children’s Study, National Center for Child Health and Development, Tokyo, Japan; c Department of Occupational and Environmental Health, Graduated School of Medical Sciences, Nagoya City University, Aichi, Japan; d Division of General Allergy, Bantane Hospital, Fujita Health University, Aichi, Japan

**Keywords:** Atopic dermatitis, birth cohort, epidemiology, IgE, pollen allergy, pollen food allergy syndrome, rhinitis

## Abstract

**Background:**

Allergic rhinitis and pollen sensitization typically increase with age; however, longitudinal data on the prevalence of pollen–food allergy syndrome (PFAS) among Japanese adolescents are limited.

**Objective:**

We assessed the prevalence, causal foods, and sensitization status of PFAS among 17-year-olds and explored its association with comorbid allergic conditions.

**Methods:**

This study was conducted as part of the Tokyo Child Health, Disease, and Development Research, a prospective birth cohort study involving the general population. Adolescents aged 17 (range, 16-18) years participated in a cross-sectional survey that included a medical history and health questionnaire, alongside serum IgE testing by ImmunoCAP ISAC. Statistical analyses were performed by descriptive statistics.

**Results:**

Among 458 participants, 54.4% had current pollen allergy and 11.2% had PFAS. The most common causal foods were apples (45.1%), kiwis (41.2%), and pineapples (39.2%). Sensitization rates were high for Cry j 1 (96.1%), Bet v 1 (70.6%), Mal d 1 (64.7%), and Pru p 1 (62.7%). Additionally, 43.1% of adolescents with PFAS had a history of atopic dermatitis, suggesting a link between PFAS and the concept of the allergic march. Rhinitis symptoms peaked in spring, with 79.8% reporting symptoms, particularly in March and April.

**Conclusion:**

This study examined the prevalence and sensitization status of PFAS among Japanese adolescents. PFAS was common in those with pollen allergies and was associated with atopic dermatitis, supporting the allergic march hypothesis. Apples, kiwis, and pineapples were the most frequently implicated foods. These findings underscore the importance of recognizing PFAS in managing adolescent allergic conditions.

## Graphical abstract

**Figure:**
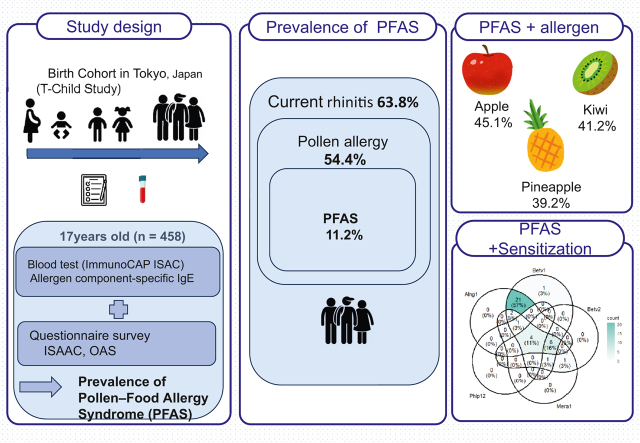

Pollen–food allergy syndrome (PFAS), also known as oral allergy syndrome (OAS), is an IgE-mediated allergic reaction that occurs in individuals with pollen allergies when they consume certain plant-derived foods.^1^ PFAS is characterized by symptoms primarily affecting the oropharynx, such as itching and swelling, and in some cases, it can lead to systemic reactions such as anaphylaxis. The prevalence of PFAS varies geographically and demographically, influenced by regional pollen types and dietary habits. Pan-allergens, such as profilins, PR-10 proteins, and lipid transfer proteins, are responsible for the cross-reactivity in PFAS.^2^ These proteins are found in both pollens and plant-derived foods.

The prevalence of allergic rhinitis has been increasing in multiple regions worldwide. In a previous study, we observed that the prevalence allergic rhinitis increased from approximately 11% at age 5 to approximately 31% by age 9 in Japan.^3^ In another study, the prevalence at age 13 was 68.8%, suggesting a continued upward trend.^4^ Cry j 1, a major allergen derived from Japanese cedar *(Cryptomeria japonica)* pollen, plays a crucial role in cedar pollen–induced allergic rhinitis in Japan. Given that the prevalence of allergic rhinitis, including sensitization to pollens, generally increases with age, the incidence of PFAS may also increase accordingly. We previously reported that the prevalence of PFAS among adolescents aged 13 years in the general population was approximately 10%.^4^ To our knowledge, no epidemiologic studies have assessed temporal changes in PFAS prevalence within the general population in Japan, particularly among 17-year-old adolescents. This study aimed to examine whether there were any changes in PFAS prevalence over a follow-up period of 5 years.

## Methods

### Study design, setting, and participants

This cross-sectional study of 17-year-old adolescents (range, 16-18 years) was conducted as part of the Tokyo Children’s Health, Illness, and Development study (T-Child study), a prospective birth cohort study of the general population conducted by the National Center for Child Health and Development.^5^, ^6^, ^7^, ^8^, ^9^, ^10^ The study aims to investigate various factors influencing child health and development, particularly in relation to allergic diseases. Newborns born between March 2004 and August 2006 were enrolled. The comprehensive methodology has been detailed in previous publications.^5^, ^6^, ^7^, ^8^, ^9^, ^10^ In the present study, 458 adolescents aged 17 years underwent serum sampling and completed questionnaire surveys.

### Ethics statement

The T-Child study was approved by the National Center for Child Health and Development research ethics committee (approval 52) and complies with the ethical standards outlined in the Japanese guidelines for medical research involving human participants (Ministry of Health, Labour and Welfare) as well as the Declaration of Helsinki. Written informed consent was obtained from all guardians, and informed assent was obtained from all minors.

### Outcome variables

Variables related to PFAS, pollen allergies, OAS, and sensitization to IgE components are presented in Table E1 in the Online Repository available at www.jaci-global.org. In this study, *OAS* is defined as oral symptoms to fruits and vegetables as reported by participants. *Pollen allergy* is defined as the presence of rhinitis symptoms along with sensitization to plants. *PFAS* is defined as fulfilling both the criteria for OAS and pollen allergy. These definitions for late adolescents aged 17 years were consistent with those applied to 13-year-olds in our previous study.^4^^,^^8^ Rhinitis was assessed by the International Study of Asthma and Allergies in Childhood (ISAAC) questionnaire.^11^, ^12^, ^13^ Sensitization to IgE components was analyzed by ImmunoCAP ISAC.

### Questionnaire survey

Participants and their parents completed a paper-based questionnaire in Japanese, which included medical history items related to ISAAC, OAS, and PFAS, to assess the health and daily life of 17-year-olds. The ISAAC questionnaire is widely used in epidemiologic studies on asthma, allergic rhinitis, and atopic dermatitis (AD) and has been validated for cross-cultural use.^14^ Definition of allergic outcomes are shown in Table E1.

### Blood sampling and IgE component measurement

Venous blood samples were collected from the participants. Allergen component–specific IgE antibody levels were measured by the multiplex array ImmunoCAP ISAC at a private contract research laboratory (Thermo Fisher Scientific, Lillerød, Denmark).^3^^,^^15^^,^^16^ The ImmunoCAP ISAC utilizes a fixed panel of 112 allergen components from 51 sources to measure IgE levels in a single test. Sample management was handled by a private contract research laboratory (SRL, Tokyo, Japan). The ImmunoCAP ISAC test is a diagnostic tool used to identify and measure allergic sensitization to a broad range of allergens, including common environmental allergens (eg, pollen, mold, and dust mites) and food allergens.^15^ These allergens are bound to small chips, with each component representing a specific protein or molecule that can trigger an allergic response in susceptible individuals. This test is widely used in both clinical practice and research.

### Bias and study size

The study participants were 17-year-olds who underwent health assessments as part of the T-Child study, which included children from the broader general community. All adolescents had been followed since birth, well before the onset of PFAS, minimizing recall and selection bias. The cohort was representative of the general population in the Tokyo metropolitan area of Japan.

### Statistical analysis

The study population comprised children born as singletons and followed until age 17 years who had no missing values in their blood test data. Descriptive statistics were used to summarize participant characteristics and variable distributions. Continuous variables are presented as means with standard deviations or medians with interquartile ranges, depending on data distribution. Categorical variables are expressed as frequencies and percentages. Statistical calculations and analyses were performed by EZR v1.54. To illustrate the relationship between PFAS and IgE component sensitizations, Venn diagrams were generated by R v4.4.2 with the packages ‘ggplot2’ and ‘ggVennDiagram.’

## Results

### Participant characteristics

Table I presents the baseline characteristics of the study participants. Among them, 67 (14.7%) had a history of OAS and 361 (78.8%) were sensitized to one or more tree, grass, or weed allergens. Additionally, 248 adolescents (54.4%) currently had pollen allergy. PFAS was observed in 51 participants (11.2%), corresponding to 20.6% of those with pollen allergy.

**Table I:** Baseline characteristics of study participants

Characteristic	No.∗	No. (%) or mean ± SD
Adolescent baseline characteristics		
Female sex	458	250 (54.6)
Height (cm)	458	164.2 ± 8.1
Body weight (kg)	458	54.5 ± 9.0
School or job status		
Full-time educational institutions such as high schools and technical colleges	458	439 (95.9)
Non–full-time educational institutions such as part-time high schools and correspondence high schools	458	17 (3.7)
Full-time employment	458	0
Adolescent allergy outcomes		
Current eczema	455	49 (10.8)
Ever AD	458	104 (22.7)
Current AD	458	48 (10.5)
Current rhinitis	456	291 (63.8)
Current conjunctivitis	456	169 (37.1)
Current asthma	458	14 (3.1)
Current wheezing	457	27 (5.9)
Current wheezing during exercise	457	13 (2.8)
Current cough at night	457	5 (1.1)
Ever animal allergy	457	107 (23.4)
Current animal allergy	457	28 (6.1)
Ever food hypersensitivity	458	51 (11.1)
Latex allergy	458	6 (1.3)
Any allergy sensitization (IgE component sensitization)	458	395 (86.2)
OAS	455	67 (14.7)
Sensitization of trees, grass, or weeds	458	361 (78.8)
Current pollen allergy	456	248 (54.4)
PFAS	454	51 (11.2)
Environmental exposure		
Environmental tobacco smoke (at home)	458	76 (16.6)
Current pet ownership	457	129 (28.2)

∗ Number of participants without missing values.

Table II outlines the months during which participants reported rhinitis symptoms throughout the year. Among those with pollen allergy, March (67.7%), April (65.3%), May (44.8%), and February (44.4%) had the highest incidence of rhinitis symptoms. Seasonally, spring (79.8%) showed the highest occurrence of rhinitis symptoms, followed by winter (54.0%), autumn (52.0%), and summer (35.1%).

**Table II:** Seasons in which 17-year-olds exhibit symptoms of rhinitis

Characteristic	Current rhinitis	Current pollen allergy	PFAS
No. of subjects	291	248	51
Month			
January	100 (34.4)	79 (31.9)	20 (39.2)
February	130 (44.7)	110 (44.4)	29 (56.9)
March	192 (66.0)	168 (67.7)	39 (76.5)
April	184 (63.2)	162 (65.3)	36 (70.6)
May	130 (44.7)	111 (44.8)	25 (49.0)
June	83 (28.5)	67 (27.0)	15 (29.4)
July	81 (27.8)	66 (26.6)	14 (27.5)
August	79 (27.1)	63 (25.4)	13 (25.5)
September	106 (36.4)	88 (35.5)	22 (43.1)
October	125 (43.0)	103 (41.5)	26 (51.0)
November	110 (37.8)	88 (35.5)	20 (39.2)
December	100 (34.4)	77 (31.0)	18 (35.3)
Season			
Spring	229 (78.7)	198 (79.8)	44 (86.3)
Summer	107 (36.8)	87 (35.1)	18 (35.3)
Autumn	157 (54.0)	129 (52.0)	30 (58.8)
Winter	158 (54.3)	134 (54.0)	33 (64.7)

Data are presented as nos. (%).

### Characteristics of PFAS patients

The characteristics of the 51 participants with PFAS are shown in Table III. Among them, 22 (43.1%) had a history of AD and 12 (23.5%) were currently experiencing AD. Additionally, 2 (3.9%) participants were currently diagnosed with asthma, and 21 (41.2%) reported a history of food hypersensitivity (parent-reported history of doctor-diagnosed food allergy).

**Table III:** Characteristics of allergic outcomes of 17-year-old adolescents with PFAS

Characteristic	No.∗	No. (%)
Adolescent allergy outcomes		
Current rhinitis	51	51 (100)
Current conjunctivitis	50	34 (68)
OAS	51	51 (100)
Current asthma	51	2 (3.9)
Current wheezing	51	6 (11.8)
Current wheezing during exercise	51	3 (5.9)
Current cough at night	51	0 (0)
Current eczema	51	14 (27.5)
Ever AD	51	22 (43.1)
Current AD	51	12 (23.5)
Ever food hypersensitivity	51	21 (41.2)
Ever animal allergy	51	28 (54.9)
Current animal allergy	51	5 (9.8)
Latex allergy	51	2 (3.9)
Any allergy sensitization (IgE component sensitization)	51	51 (100)
Sensitization of trees, grass, or weeds	51	51 (100)
Environmental exposure		
Environmental tobacco smoke (at home)	51	8 (15.7)
Current pet ownership at 17 years	51	11 (21.6)

∗ Number of participants without missing values.

### Foods triggering PFAS

The foods associated with PFAS in this study are listed in Table IV. The most frequently implicated foods were apples (45.1%), followed by kiwis (41.2%), pineapples (39.2%), peaches (37.3%), and cherries (25.5%).

**Table IV:** Causal food allergens in PFAS

Characteristic	PFAS, no. (%)
Causal food allergen	
Apple	23 (45.1)
Kiwi fruit	21 (41.2)
Pineapple	20 (39.2)
Peach	19 (37.3)
Cherry	13 (25.5)
Melon	11 (21.6)
Plum	10 (19.6)
Soybean, processed soybean foods	9 (17.6)
Peanuts	9 (17.6)
Loquat	7 (13.7)
Pear	6 (11.8)
Mango	6 (11.8)
Walnut	6 (11.8)
Hazelnut	6 (11.8)
Strawberry	5 (9.8)
Tomato	5 (9.8)
Watermelon	4 (7.8)
Banana	4 (7.8)
Cashew nuts	4 (7.8)
Grapes	3 (5.9)
Almond	3 (5.9)
Eggplant	3 (5.9)
Persimmon	2 (3.9)
Celery	2 (3.9)
Avocado	2 (3.9)
Mandarin orange	1 (2.0)
Carrot	1 (2.0)
No. of causal food allergens	
1	11 (21.6)
2	8 (15.7)
3	9 (17.6)
4	8 (15.7)
5	5 (9.8)
6	1 (2.0)
7	2 (3.9)
8	1 (2.0)
9	2 (3.9)
10	3 (5.9)
17	1 (2.0)

### Sensitization status of PFAS

**Fig 1:**
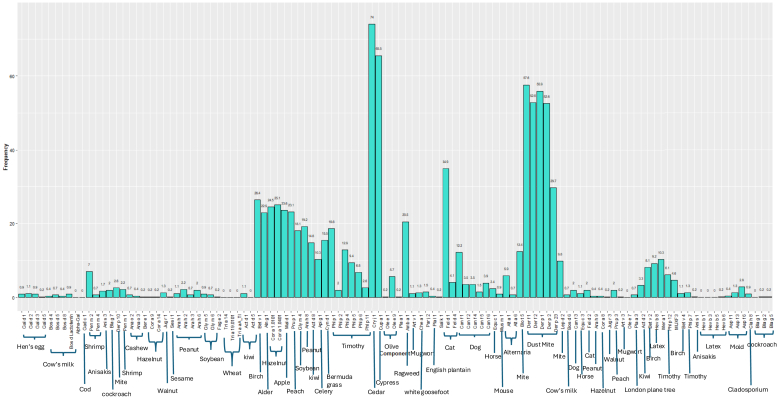
Prevalence of IgE sensitization to ImmunoCAP ISAC components among adolescents aged 17 years.

**Fig 2:**
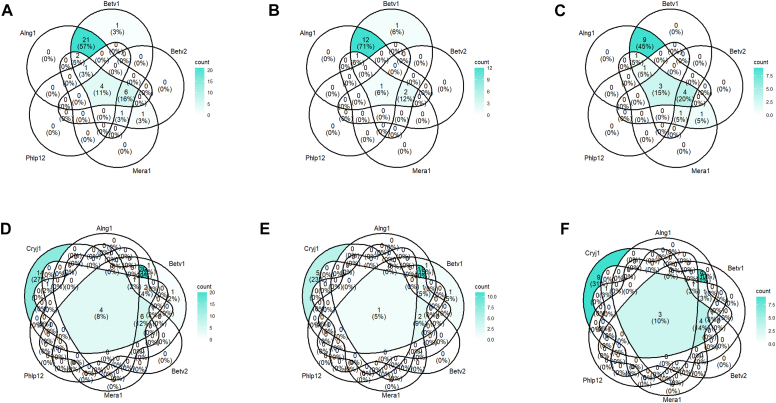
Venn diagrams of component sensitization among **(A)** adolescents with PFAS; **(B)** those with both AD and PFAS; **(C)** those with PFAS but without AD-showing sensitization to 5 components (Bet v 1, Bet v 2, Mer a 1, Phl p 12, and Aln g 1); **(D)** those with PFAS; **(E)** those with both AD and PFAS; and **(F)** those with PFAS but without AD, showing sensitization to 6 components: Bet v 1, Bet v 2, Mer a 1, Phl p 12, Aln g 1, and Cry j 1.

Fig 1 as well as Fig E1 in the Online Repository available at www.jaci-global.org show IgE component sensitization among all study participants. The respiratory and food sensitization profiles of adolescents with PFAS are presented in Tables V and VI. Sensitization rates were notably high for several allergens: Cry j 1 (Japanese cedar, 96.1%), Der f 1 (house dust mite, 84.3%), Bet v 1 (birch, 70.6%), and Fel d 1 (cat, 70.6%). Additionally, over half of the adolescents with PFAS were sensitized to Cor a 1.0401 (hazelnut, 70.6%), Ara h 8 (peanut, 60.8%), Mal d 1 (apple, 64.7%), and Pru p 1 (peach, 62.7%). Fig 2 as well as Figs E2-E7 in the Online Repository display Venn diagrams of component sensitizations (Bet v 1, Bet v 2, Mer a 1, Phl p 12, and Aln g 1) among all participants with PFAS, those with PFAS and AD, and those with PFAS without AD.

**Table V:** Inhalant allergen component sensitization by allergic outcomes at 17 years old

Component	Allergen	Current rhinitis (n = 291)	OAS (n = 67)	Current pollen allergy (n = 248)	Pollen food allergy syndrome (n = 51)
Aln g 1	Alder	75 (25.8)	40 (59.7)	75 (30.2)	34 (66.7)
Amb a 1	Short ragweed	69 (23.7)	20 (29.9)	69 (27.8)	17 (33.3)
Art v 1	Mugwort	3 (1.0)	3 (4.5)	3 (1.2)	2 (3.9)
Art v 3	Mugwort	0	0	0	0
Bet v 1	Birch	87 (29.9)	42 (62.7)	87 (35.1)	36 (70.6)
Bet v 2	Birch	25 (8.6)	13 (19.4)	25 (10.1)	12 (23.5)
Bet v 4	Birch	2 (0.7)	2 (3.0)	2 (0.8)	1 (2.0)
Che a 1	Lamb’s quarters	6 (2.1)	1 (1.5)	6 (2.4)	1 (2.0)
Cry j 1	Japanese cedar	235 (80.8)	62 (92.5)	235 (94.8)	49 (96.1)
Cup a 1	Cypress	208 (71.5)	59 (88.1)	208 (83.9)	47 (92.9)
Cyn d 1	Bermuda grass	48 (16.5)	12 (17.9)	48 (19.4)	9 (17.6)
Mer a 1	Annual mercury	34 (11.7)	16 (23.9)	34 (13.7)	15 (29.4)
Ole e 1	Olive	1 (0.3)	1 (1.5)	1 (0.4)	1 (2.0)
Ole e 7	Olive	0	0	0	0
Ole e 9	Olive	20 (6.9)	7 (10.4)	20 (8.1)	6 (11.8)
Par j 2	Pellitory of the wall	6 (2.1)	2 (3.0)	6 (2.4)	2 (3.9)
Phl p 1	Timothy	55 (18.9)	16 (23.9)	55 (22.2)	12 (23.5)
Phl p 11	Timothy	9 (3.1)	4 (6.0)	9 (3.6)	3 (5.9)
Phl p 12	Timothy	18 (6.2)	5 (7.5)	18 (7.3)	5 (9.8)
Phl p 2	Timothy	6 (2.1)	3 (4.5)	6 (2.4)	2 (3.9)
Phl p 4	Timothy	41 (14.1)	14 (20.9)	41 (16.5)	12 (23.5)
Phl p 5	Timothy	29 (10.0)	9 (13.4)	29 (11.7)	8 (15.7)
Phl p 6	Timothy	22 (7.6)	7 (10.4)	22 (8.9)	7 (13.7)
Phl p 7	Timothy	3 (1.0)	3 (4.5)	3 (1.2)	2 (3.9)
Pla a 1	London plane	1 (0.3)	1 (1.5)	1 (0.4)	1 (2.0)
Pla a 3	London plane	2 (0.7)	2 (3.0)	2 (0.8)	2 (3.9)
Pla l 1	English plantain	2 (0.7)	1 (1.5)	2 (0.8)	1 (2.0)
Sal k 1	Saltwort	1 (0.3)	0	1 (0.4)	0
Der f 1	American HDM	186 (63.9)	52 (77.6)	178 (71.8)	43 (84.3)
Der f 2	American HDM	173 (59.5)	45 (67.2)	163 (65.7)	37 (72.5)
Der p 1	European HDM	181 (62.2)	48 (71.6)	174 (70.2)	41 (80.4)
Der p 10	European HDM	9 (3.1)	2 (3.0)	8 (3.2)	1 (2.0)
Der p 2	European HDM	172 (59.1)	45 (67.2)	162 (65.3)	37 (72.5)
Der p 23	European HDM	96 (33.0)	22 (32.8)	91 (36.7)	15 (29.4)
Can f 1	Domestic dog	40 (13.7)	13 (19.4)	39 (15.7)	9 (17.6)
Can f 2	Domestic dog	11 (3.8)	3 (4.5)	11 (4.4)	3 (5.9)
Can f 3	Domestic dog	7 (2.4)	5 (7.5)	7 (2.8)	4 (7.8)
Can f 4	Domestic dog	14 (4.8)	2 (3.0)	13 (5.2)	2 (3.9)
Can f 5	Domestic dog	5 (1.7)	4 (6.0)	5 (2.0)	3 (5.9)
Can f 6	Domestic dog	12 (4.1)	1 (1.5)	10 (4.0)	1 (2.0)
Fel d 1	Domestic cat	118 (40.5)	41 (61.2)	116 (46.8)	36 (70.6)
Fel d 2	Domestic cat	6 (2.1)	3 (4.5)	6 (2.4)	2 (3.9)
Fel d 4	Domestic cat	13 (4.5)	3 (4.5)	12 (4.8)	2 (3.9)
Equ c 1	Domestic horse	9 (3.1)	3 (4.5)	8 (3.2)	3 (5.9)
Equ c 3	Domestic horse	4 (1.4)	3 (4.5)	4 (1.6)	2 (3.9)
Alt a 1	*Alternaria alternata*	16 (5.5)	4 (6.0)	15 (6.0)	3 (5.9)
Alt a 6	*A alternata*	3 (1.0)	1 (1.5)	2 (0.8)	1 (2.0)
Asp f 1	*Aspergillus fumigatus*	2 (0.7)	0	2 (0.8)	0
Asp f 3	*A fumigatus*	4 (1.4)	2 (3.0)	4 (1.6)	2 (3.9)
Asp f 6	*A fumigatus*	10 (3.4)	3 (4.5)	10 (4.0)	2 (3.9)
Cla h 8	*Cladosporium herbarum*	2 (0.7)	0	2 (0.8)	0

Data are presented as nos. (%).

*HDM,* House dust mite.

**Table VI:** Food allergen component sensitization by allergic outcomes in 17-year-olds

Component	Allergen	Current rhinitis (n = 291)	OAS (n = 67)	Current pollen allergy (n = 248)	PFAS (n = 51)
Act d 1	Kiwi	5 (1.7)	3 (4.5)	5 (2.0)	3 (5.9)
Act d 2	Kiwi	8 (2.7)	3 (4.5)	8 (3.2)	3 (5.9)
Act d 5	Kiwi	0	0	0	0
Act d 8	Kiwi	50 (17.2)	30 (44.8)	50 (20.2)	25 (49.0)
Ana o 2	Cashew	3 (1.0)	1 (1.5)	3 (1.2)	1 (2.0)
Ana o 3	Cashew	2 (0.7)	1 (1.5)	2 (0.8)	1 (2.0)
Api g 1	Celery	34 (11.7)	18 (26.9)	34 (13.7)	16 (31.4)
Ara h 1	Peanut	4 (1.4)	5 (7.5)	4 (1.6)	4 (7.8)
Ara h 2	Peanut	9 (3.1)	9 (13.4)	9 (3.6)	8 (15.7)
Ara h 3	Peanut	3 (1.6)	3 (4.5)	3 (1.2)	3 (5.9)
Ara h 6	Peanut	8 (2.7)	7 (10.4)	8 (3.2)	6 (11.8)
Ara h 8	Peanut	65 (22.3)	37 (55.2)	65 (26.2)	31 (60.8)
Ara h 9	Peanut	0	0	0	0
Ber e 1	Brazil nut	1 (0.3)	1 (1.5)	1 (0.4)	1 (2.0)
Cor a 1.0101	Hazelnut	81 (27.8)	39 (58.2)	81 (32.7)	33 (64.7)
Cor a 1.0401	Hazelnut	85 (29.2)	42 (62.7)	85 (34.3)	36 (70.6)
Cor a 8	Hazelnut	1 (0.3)	0	1 (0.4)	0
Cor a 9	Hazelnut	1 (0.3)	1 (1.5)	1 (0.4)	1 (2.0)
Cor a 14	Hazelnut	0	1 (1.5)	0	0
Gly m 4	Soybean	61 (21.0)	34 (50.7)	61 (24.6)	29 (56.9)
Gly m 5	Soybean	2 (0.7)	1 (1.5)	2 (0.8)	1 (2.0)
Gly m 6	Soybean	3 (1.0)	1 (1.5)	3 (1.2)	1 (2.0)
Jug r 1	Walnut	5 (1.7)	5 (7.5)	5 (2.0)	4 (7.8)
Jug r 3	Walnut	0	0	0	0
Mal d 1	Apple	80 (27.5)	39 (58.2)	80 (32.3)	33 (64.7)
Pru p 1	Peach	78 (26.8)	38 (56.7)	78 (31.5)	32 (62.7)
Pru p 3	Peach	7 (2.4)	4 (6.0)	7 (2.8)	4 (2.0)
Ses i 1	Sesame	1 (0.3)	1 (1.5)	1 (0.4)	1 (2.0)

Data are presented as nos. (%).

## Discussion

To our knowledge, this is the first study to evaluate temporal changes in PFAS using data from a general population cohort of adolescents in Japan. This study assessed 17-year-olds, among whom 54.4% had pollen allergy and 11.2% had PFAS. Among participants with PFAS, 43.1% had a history of AD. PFAS often coexists with AD during adolescence, suggesting that it contributes to the progression of the allergic march.^10^^,^^17^ The most common foods triggering PFAS included apples, kiwis, pineapples, peaches, and cherries. Seasonal rhinitis symptoms were most prominent in spring, with 79.8% of participants with pollen allergy reporting symptoms, peaking in March and April.

### Pollen allergy

Pollen allergy affected 54.4% of the 17-year-old participants in this study, compared with 51.0% in our previous study of 13-year-olds,^4^ suggesting a similar prevalence with age; however, the increase was not pronounced. Among 17-year-olds with pollen allergy, sensitization to cedar pollen was the most common (94.8%). Similarly, in the survey conducted at age 13 years, cedar pollen showed the highest sensitization rate among adolescents with pollen allergy (95.7%),^4^ indicating that cedar pollen consistently shows high sensitization rates in this population. A previous study in Japan suggested that cedar pollen allergy affects 49.5% of children aged 10-19 years, and cedar pollen has been identified as a major contributor to allergic rhinitis.^18^ The high sensitization rate to cedar pollen in the present study aligns with those findings. In contrast, a South Korean study reported that sensitization to birch and alder pollen is most common.^19^ Birch is a major cause of seasonal allergic rhinitis in Northern and Central Europe. In Germany, a study involving 260 patients with tree pollen allergies reported that 239 (92%) were sensitized to Bet v 1.^20^ Compared with these international reports, the present study found a relatively low sensitization rate to birch pollen. In Japan, birch trees are primarily distributed in specific regions, such as Hokkaido, unlike cedar pollen, which is more widespread. Given that this study focused on children living in Tokyo, sensitization to birch pollen is likely limited in this population.

When comparing our previous study of 13-year-olds with the T-Child study,^4^ no significant changes were found in sensitization rates to birch and alder pollen among adolescents with pollen allergy. Similarly, no significant changes were observed in sensitization rates to weeds such as ragweed and grasses such as timothy grass.

### PFAS in adolescents

In this study of 17-year-olds, 11.2% had PFAS, and among those with pollen allergy, 20.6% had PFAS. In our previous study of 13-year-olds, 11.7% had PFAS, and 22.9% of those with pollen allergy were affected.^4^ The prevalence of PFAS has remained relatively stable since the age of 13 years, suggesting a low likelihood of a significant increase during puberty. In Europe, kiwi and peach are the most frequently identified causative foods,^21^ whereas in our Japanese cohort, apple was the most common, followed by kiwi. Although kiwi showed a consistently high frequency in both Europe and Japan, regional differences in causative pollen likely influence the associated PFAS-triggering foods, suggesting that PFAS exhibits region-specific characteristics.

### Sensitization status of PFAS in adolescents

In this study, Cry j 1 (96.1%) was the most common allergen responsible for sensitization among adolescents with PFAS, consistent with our previous findings at age 13 years,^4^ wherein the sensitization rate to Cry j 1 was 93.2%. This indicates that sensitization to Cry j 1 remained stable over time. Conversely, sensitization rates to Bet v 1 and Aln g 1 among adolescents with PFAS increased from 13 to 17 years of age; specifically, sensitization Bet v 1 increased from 59.3% to 70.6% and that to Aln g 1 from 47.5% to 66.7% . Additionally, increases were observed in the sensitization rates to other allergens, including Mal d 1 (from 55.9% to 64.7%), Pru p 1 (from 54.2% to 62.7%), Gly m 4 (from 40.7% to 56.9%), Cor a 1.0101 (from 45.8% to 64.7%), and Cor a 1.0401 (from 50.8% to 70.6%). These increased sensitization rates may be attributed to a “molecular spreading” process, wherein sensitization expands from the preclinical to the clinical stage.^22^

The causative foods for PFAS also changed between the two survey periods. At age 13 years, kiwi, pineapple, and apple were the primary foods implicated, whereas at age 17 years, apple became the leading causative food, followed by kiwi and pineapple.

Bet v 1 is a PR-10 protein found in birch pollen, and its homologs, which exhibit cross-reactivity, are present in various foods from the Rosaceae and Fabaceae families. Common examples include Mal d 1 (apple), Pru p 1 (peach), and Gly m 4 (soybean).^23^^,^^24^ Additionally, Bet v 1 and Aln g 1 (from alder pollen) share over 80% protein homology,^25^ which may explain the increased sensitization rates to these allergens among patients with PFAS as they age.

Interestingly, no significant changes were observed in the sensitization rates for Bet v 1 and Aln g 1 among adolescents with pollen allergy. At age 13 years,^4^ the sensitization rates for Bet v 1 and Aln g 1 were 36.0% and 28.3%, respectively, compared with 35.1% and 30.2% at age 17 years. These findings suggest that the overall sensitization rate for Bet v 1 among adolescents with pollen allergy does not significantly increase with age. However, among those already sensitized to Bet v 1, the increasing prevalence of PFAS may be associated with greater reactivity to Rosaceae foods, such as apples, as age increases.

### Limitations

This study of a birth cohort conducted in a nonhospital setting provided an opportunity to assess healthy adolescents who typically do not seek medical attention. Additionally, their allergen sensitization status was evaluated across a broad range. Both of these are strengths of the study.

However, this study also has several limitations. Due to its non–hospital-based setting, a comprehensive evaluation of participants’ medical histories was not feasible. Allergen component–specific IgE antibody levels were assessed by the multiplex array ImmunoCAP ISAC. Consequently, the analysis was limited to the components included in the ImmunoCAP ISAC panel, and not all pan-allergen families (PR-10 proteins, profilins, lipid transfer proteins, gibberellin-regulated proteins, etc) could be evaluated.

### Conclusion

This study provides valuable insights into the temporal trends and characteristics of PFAS among Japanese adolescents. On the basis of data from a general population cohort, PFAS affected approximately 11.2% of 17-year-olds, with a notable association between PFAS and a history of AD. The findings highlight apples, kiwis, pineapples, peaches, and cherries as the most common trigger foods, suggesting a potential shift in dietary triggers with age. Additionally, the high prevalence of seasonal rhinitis symptoms during spring underscores the significant impact of pollen allergy in this population. These results emphasize the importance of monitoring PFAS prevalence and clinical presentation to guide effective prevention and management strategies.

**Clinical implication**

PFAS is common among Japanese adolescents with pollen allergy; is linked to AD; and is triggered by apples, kiwis, and pineapples, highlighting its relevance in allergy management.

## Disclosure statement

Supported by a grant from the 10.13039/100007786National Center for Child Health and Development (grant 2019E-1) and 10.13039/501100001691Japan Society for the Promotion of Science Grants-in-Aid for Scientific Research (KAKENHI; grant 22K10545). English-language editing was supported by Grammarly.

Data availability statement: The data used in this study cannot be made publicly available due to ethical considerations and Japanese privacy laws. All inquiries regarding data access should be directed to the Research Office of the Allergy Center, National Center for Child Health and Development, Tokyo, Japan (allergy_research@ncchd.go.jp).

Disclosure of potential conflict of interest: The authors declare that they have no relevant conflicts of interest.

## Supplementary data

Supplementary Data

Supplementary Table E1
