# Supplementary material for: Prevalence and sensitization of pollen–food allergy syndrome among adolescents in Tokyo
Source: J Allergy Clin Immunol Glob. 2025 Aug 28;4(4):100561. doi: 10.1016/j.jacig.2025.100561 (PMC12483659; doi:10.1016/j.jacig.2025.100561)

Online Repository

**Figure E1. Prevalence of IgE sensitization to ISAC components among adolescents at 17 years old (order by frequency).**

**
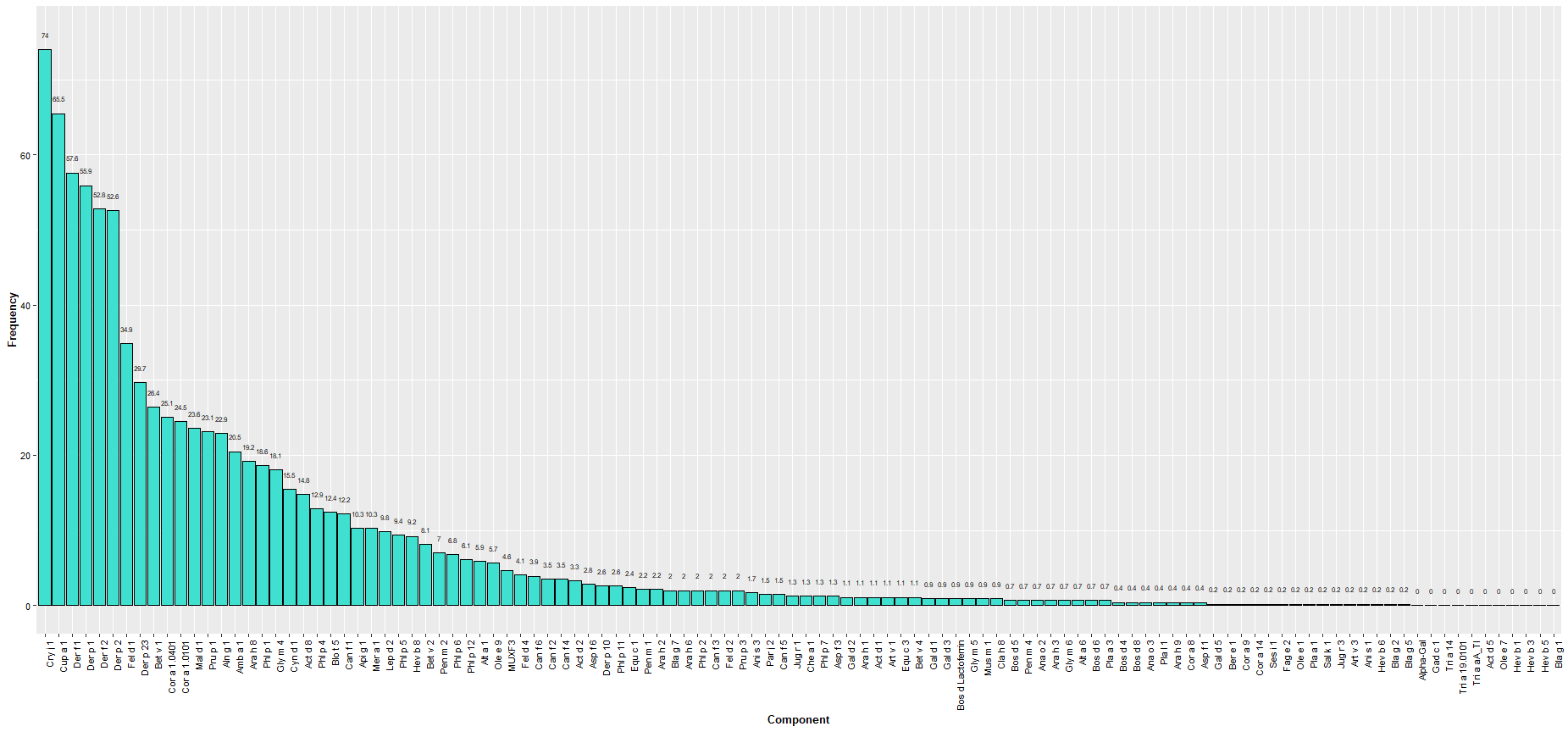
**

**Figure E2. Venn diagram of 5 component sensitization (Bet v 1, Bet v 2. Mer a 1, Phl P 12, Aln g 1) among (A) patients with PFAS triggered by Rosaceae families, (B) patients with AD and PFAS triggered by Rosaceae families, (C) patients with PFAS triggered by Rosaceae families but without AD, and 6 component sensitization (Bet v 1, Bet v 2. Mer a 1, Phl P 12, Aln g 1, Cry j 1) among (D) patients with PFAS triggered by Rosaceae families, (E) patients with AD and PFAS triggered by Rosaceae families, (F) patients with PFAS triggered by Rosaceae families but without AD.**

**
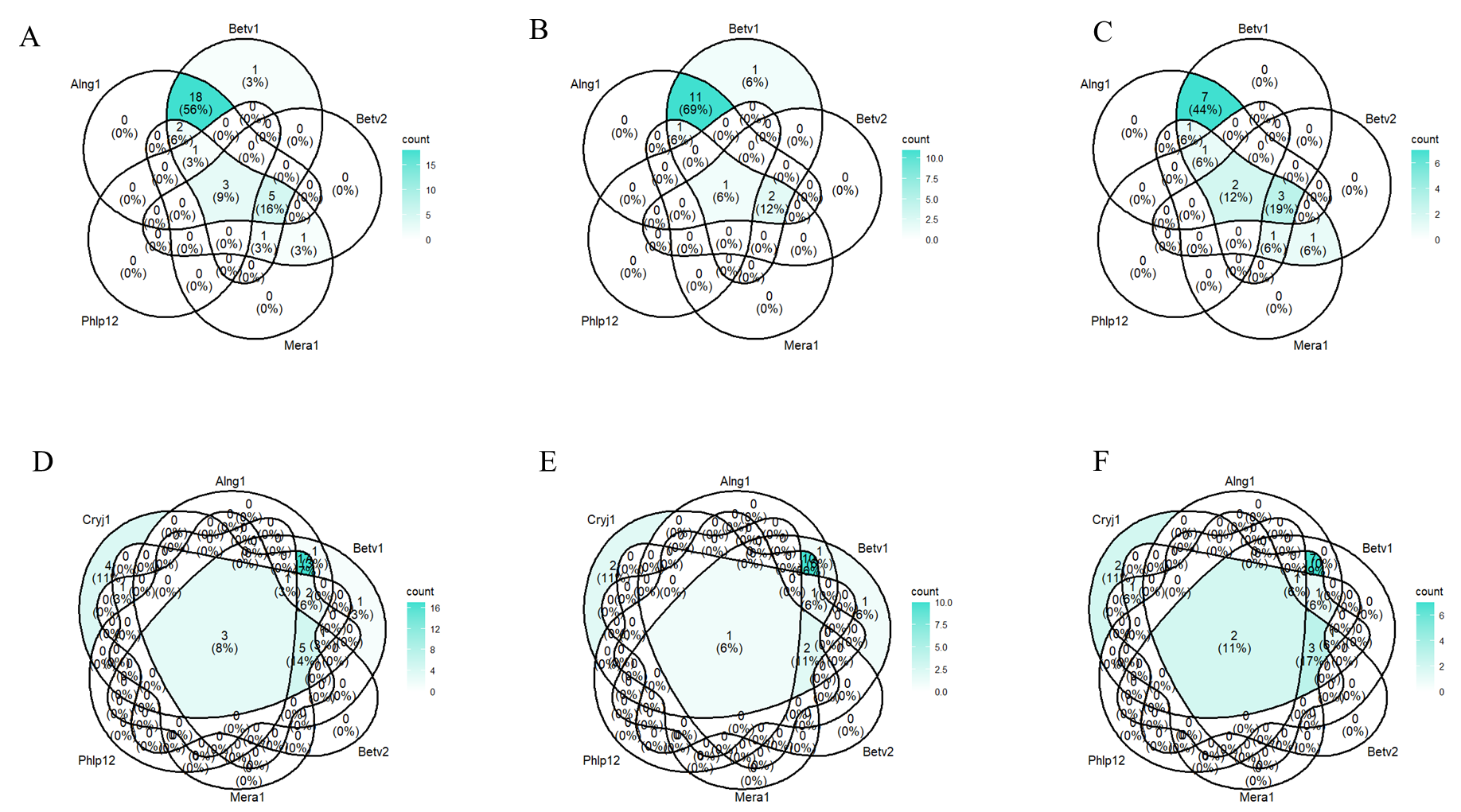
**

**Figure E3. Venn diagram of 5 component sensitization (Bet v 1, Bet v 2. Mer a 1, Phl P 12, Aln g 1) among (A) patients with PFAS triggered by Actinidiaceae families, (B) patients with AD and PFAS triggered by Actinidiaceae families, (C) patients with PFAS triggered by Actinidiaceae families but without AD, and 6 component sensitization (Bet v 1, Bet v 2. Mer a 1, Phl P 12, Aln g 1, Cry j 1) among (D) patients with PFAS triggered by Actinidiaceae families, (E) patients with AD and PFAS triggered by Actinidiaceae families, (F) patients with PFAS triggered by Actinidiaceae families but without AD.**

**
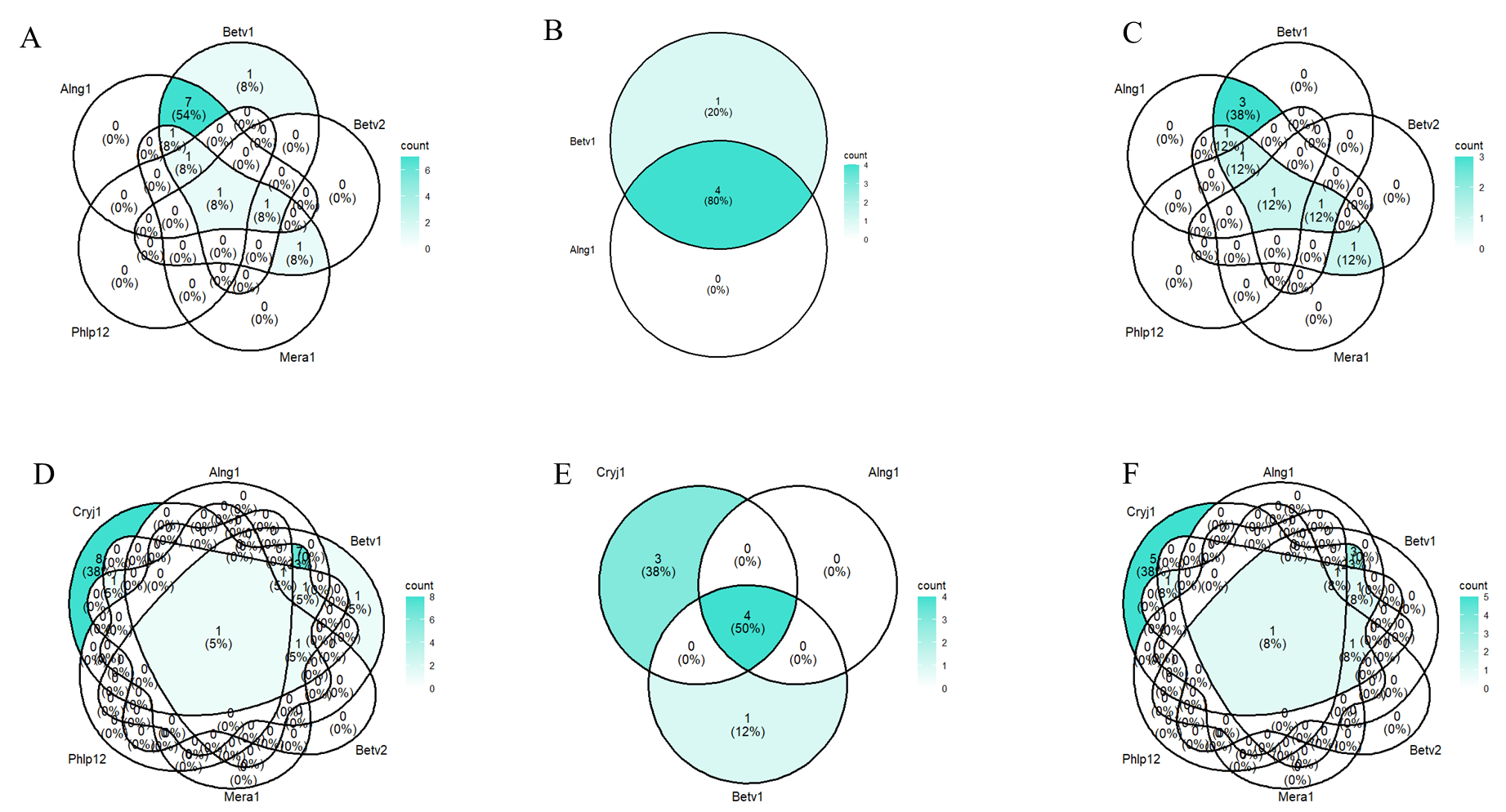
**

**Figure E4. Venn diagram of 5 component sensitizations (Bet v 1, Bet v 2. Mer a 1, Phl P 12, Aln g 1) among (A) patients with PFAS triggered by Bromeliaceae families, (B) patients with AD and PFAS triggered by Bromeliaceae families, (C) patients with PFAS triggered by Bromeliaceae families but without AD, and 6 component sensitizations (Bet v 1, Bet v 2. Mer a 1, Phl P 12, Aln g 1, Cry j 1) among (D) patients with PFAS triggered by Bromeliaceae families, (E) patients with AD and PFAS triggered by Bromeliaceae families, (F) patients with PFAS triggered by Bromeliaceae families but without AD.**

**
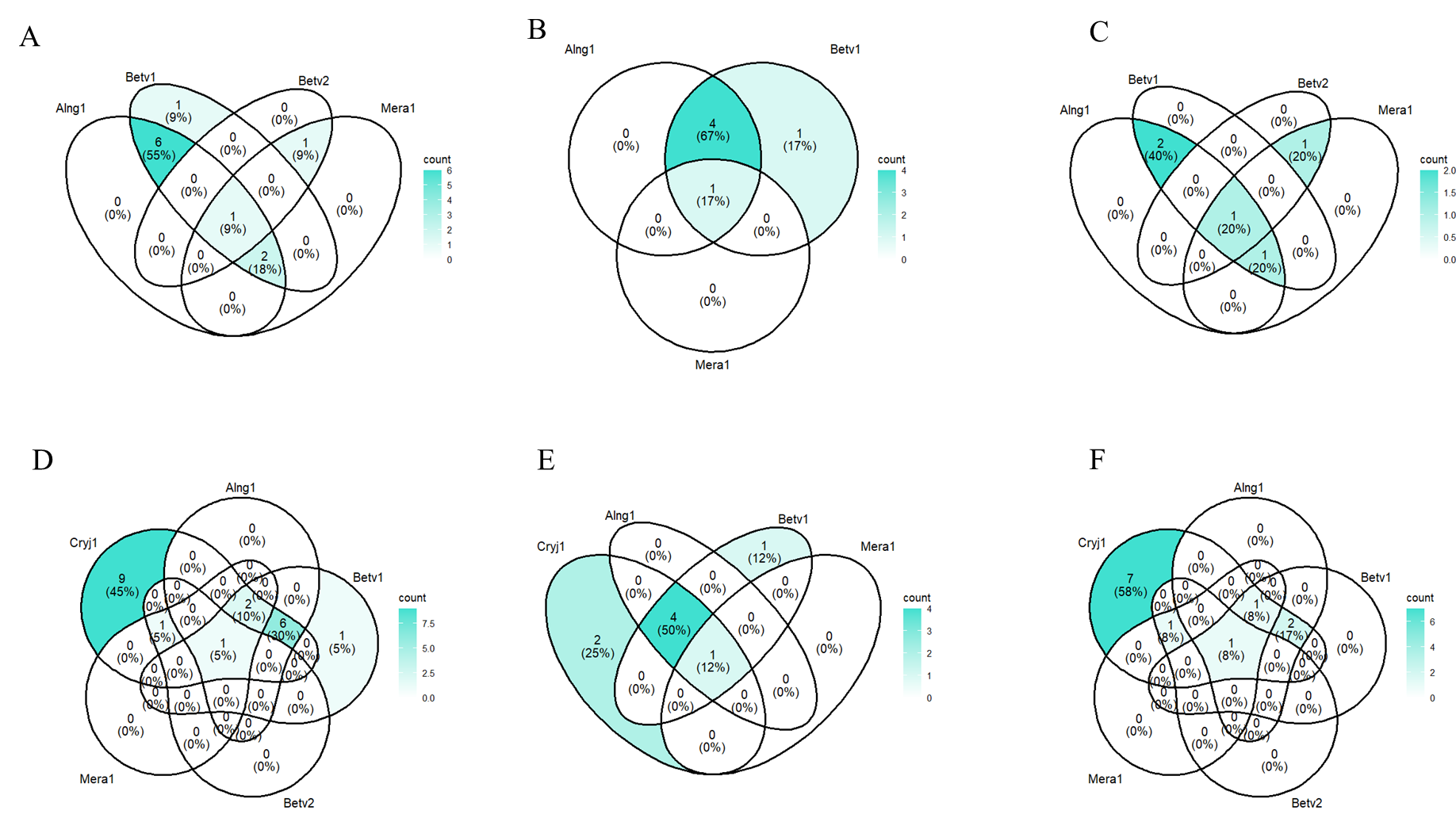
**

**Figure E5. Venn diagram of 5 component sensitizations (Bet v 1, Bet v 2. Mer a 1, Phl P 12, Aln g 1) among (A) patients with PFAS triggered by Cucurbitaceae families, (B) patients with AD and PFAS triggered by Cucurbitaceae families, (C) patients with PFAS triggered by Cucurbitaceae families but without AD, and 6 component sensitizations (Bet v 1, Bet v 2. Mer a 1, Phl P 12, Aln g 1, Cry j 1) among (D) patients with PFAS triggered by Cucurbitaceae families, (E) patients with AD and PFAS triggered by Cucurbitaceae families, (F) patients with PFAS triggered by Cucurbitaceae families but without AD.**


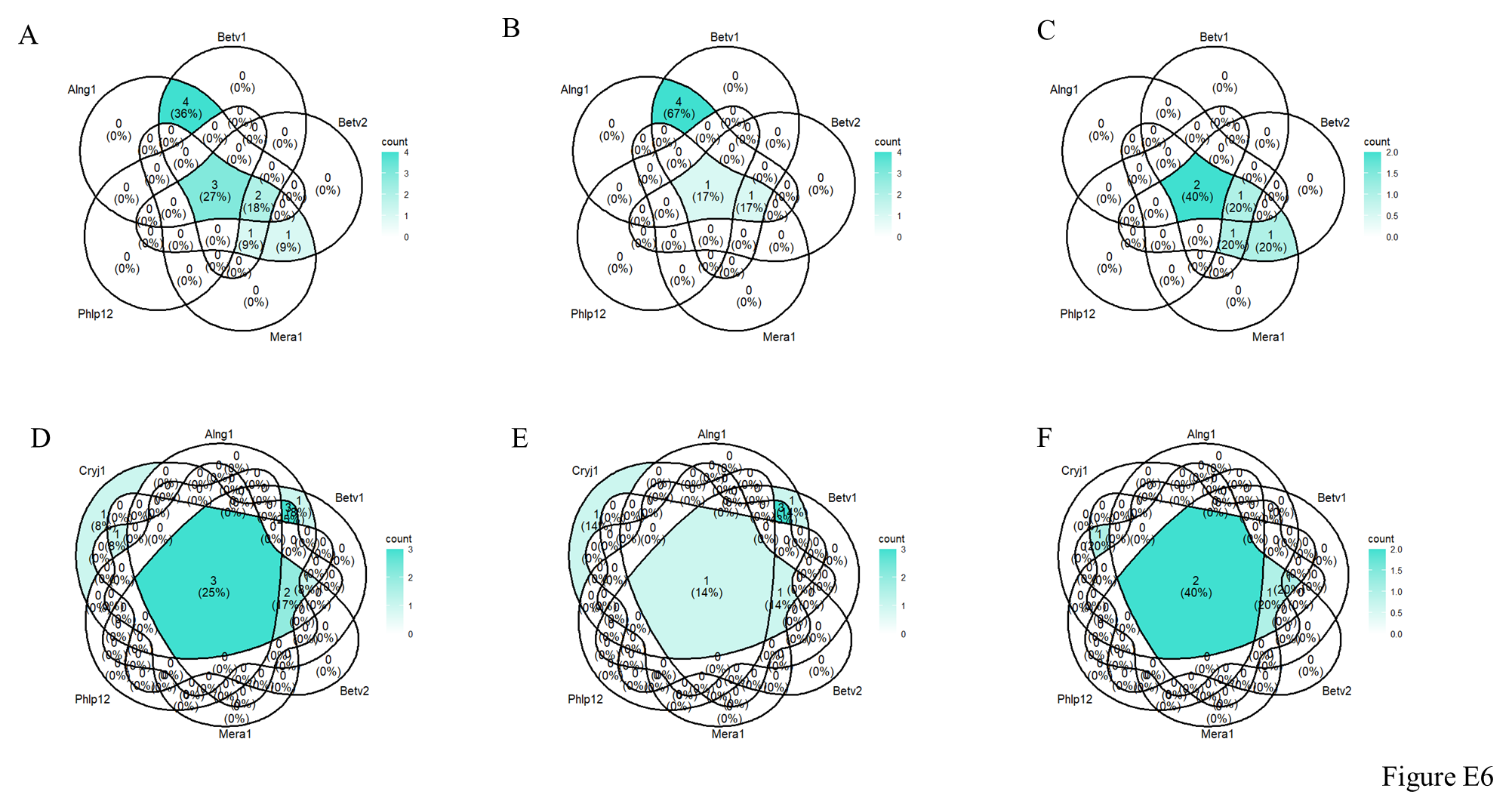


**Figure E6. Venn diagram of 5 component sensitization (Bet v 1, Bet v 2. Mer a 1, Phl P 12, Aln g 1) among (A) patients with PFAS triggered by Fabaceae families, (B) patients with AD and PFAS triggered by Fabaceae families, (C) patients with PFAS triggered by Fabaceae families but without AD, and 6 component sensitization (Bet v 1, Bet v 2. Mer a 1, Phl P 12, Aln g 1, Cry j 1) among (D) patients with PFAS triggered by Fabaceae families, (E) patients with AD and PFAS triggered by Fabaceae families, (F) patients with PFAS triggered by Fabaceae families but without AD.**

**
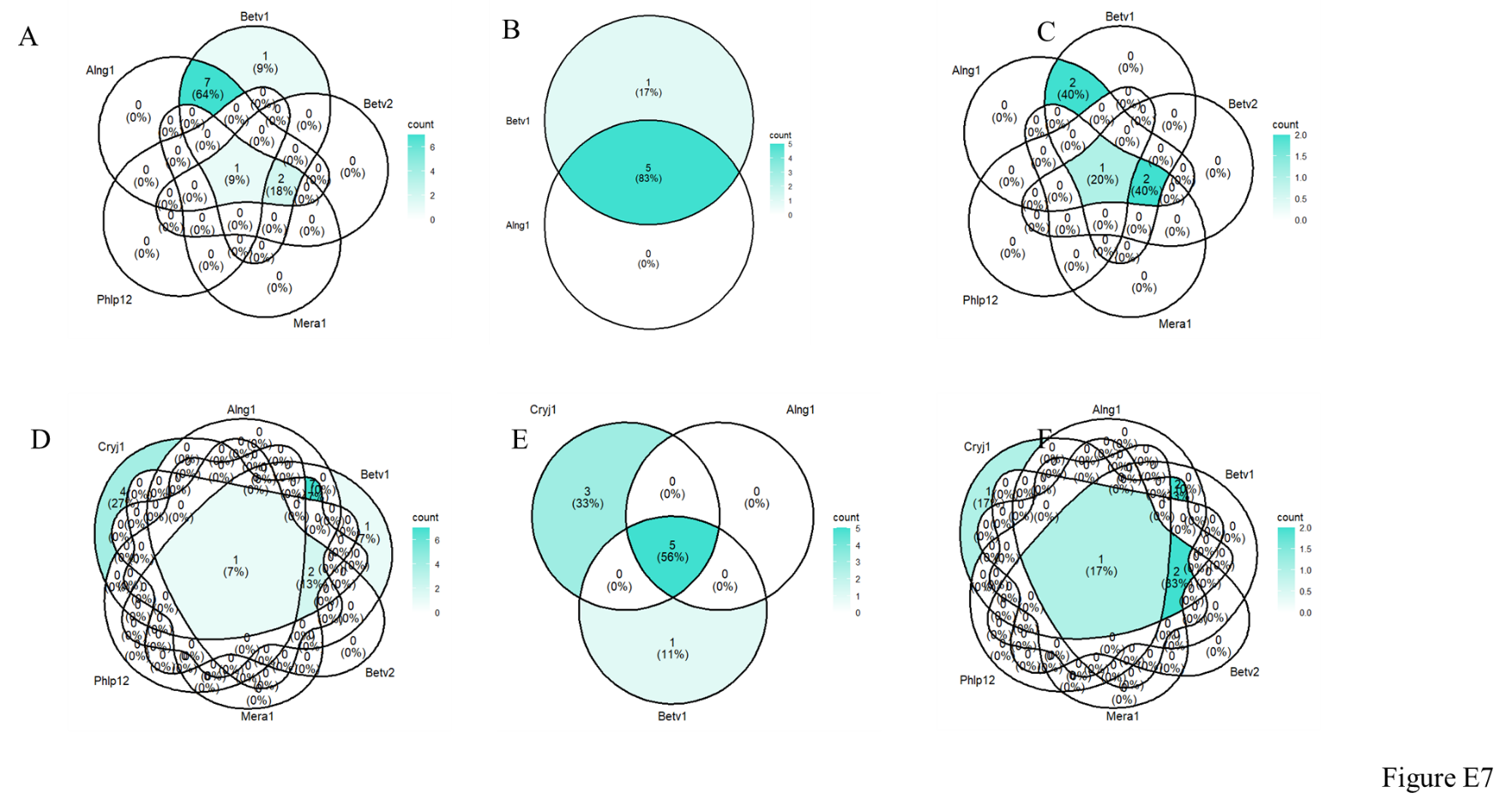
**

**Figure E7. Venn diagram of 5 component sensitization (Bet v 1, Bet v 2. Mer a 1, Phl P 12, Aln g 1) among (A) patients with PFAS triggered by Anacardiaceae families, (B) patients with AD and PFAS triggered by Anacardiaceae families, (C) patients with PFAS triggered by Anacardiaceae families but without AD, and 6 component sensitization (Bet v 1, Bet v 2. Mer a 1, Phl P 12, Aln g 1, Cry j 1) among (D) patients with PFAS triggered by Anacardiaceae families, (E) patients with AD and PFAS triggered by Anacardiaceae families, (F) patients with PFAS triggered by Anacardiaceae families but without AD.**


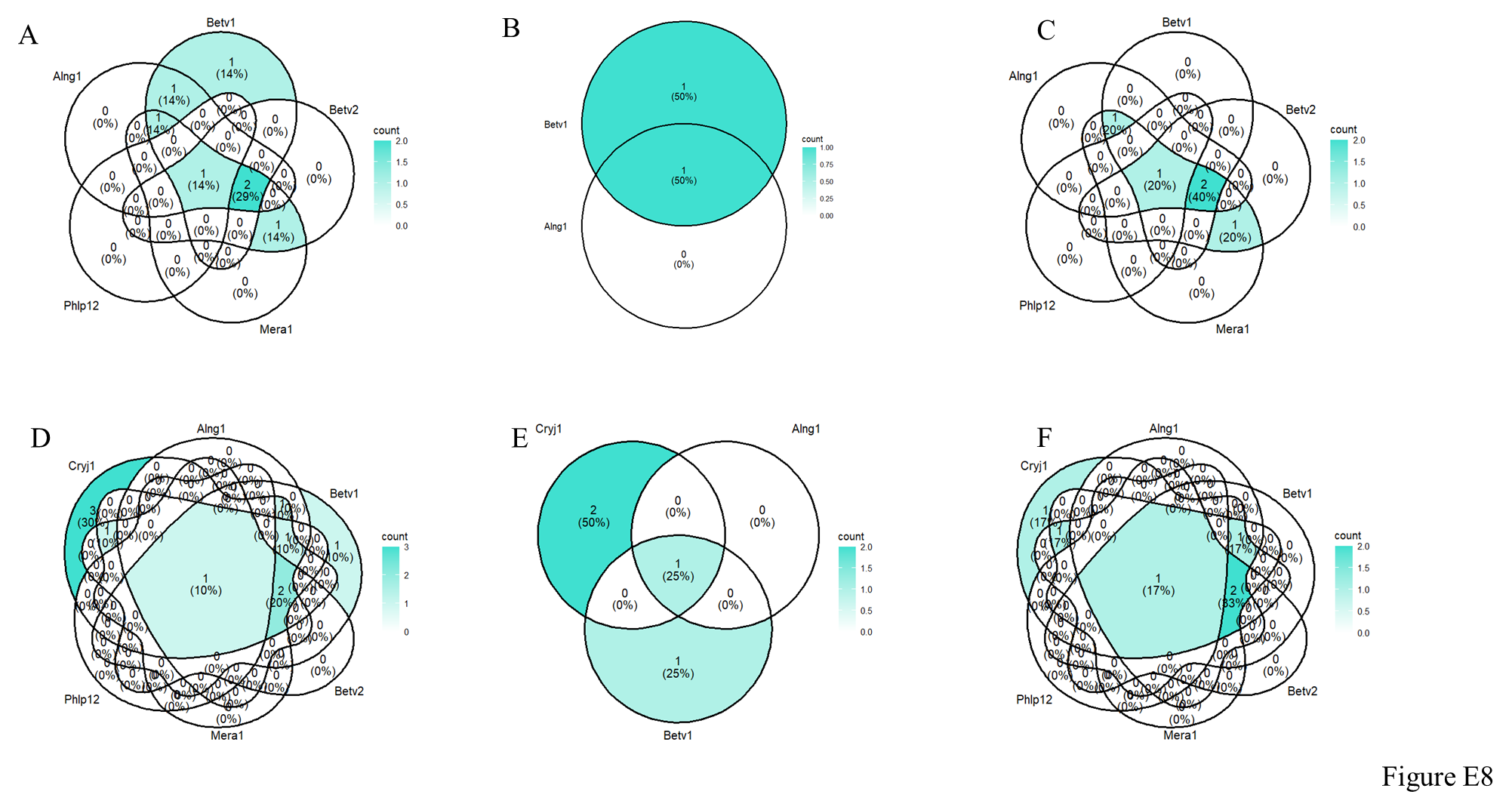

Supplement: Supplementary Data [file mmc1.docx]
