# Supplementary material for: Prevalence and sensitization of pollen–food allergy syndrome among adolescents in Tokyo
Source: J Allergy Clin Immunol Glob. 2025 Aug 28;4(4):100561. doi: 10.1016/j.jacig.2025.100561 (PMC12483659; doi:10.1016/j.jacig.2025.100561)
Supplement: Supplementary Table E1 [file mmc2.docx]

| Table E1. Definitions of Outcomes | |
| --- | --- |
| Current eczema | A positive answer from the child to the two question (child at 17y old), "Have you ever had an itchy rash which was coming and going for at least six months in the past 12 months ?" and"Has this itchy rash at any time affected any of the following places: the folds of the elbows, behind the knees, in front of the ankles, under the buttocks, or around the neck, ears or eyes?" |
| Ever atopic dermatitis | A positive answer from the caregiver to the question (child at 17y old), "Has your child ever had atopy (atopic dermatitis)?" |
| Current atopic dermatitis | A positive answer from the caregiver to the question (child at 17 y old), "Is your child currently diagnosed with atopic dermatitis by your doctor?" |
| Current rhinitis | A positive answer from the child to the question (child at 17 y old), “In the past 12 months, have you had a problem with sneezing, or a runny, or blocked nose when you did not have a cold or the flu?” |
| Current conjunctivitis | A positive answer from the child to the question (child at 17 y old), “In the past 12 months, has this nose problem been accompanied by itchy -watery eyes?" |
| Current asthma | A positive answer from the caregiver to the question (child at 17 y old), “Has your child ever been diagnosed by a doctor as having asthma in the past 12 months?” |
| Current wheeze | A positive answer from the child to the question (child at 17 y old), “Have you ever had wheezing or whistling in the past 12 months?” |
| Current wheeze during exercise | A positive answer from the child to the question (child at 17 y old),”In the past 12 months, has your chest sounded wheezy during or after exercise?” |
| Current cough at night | A positive answer from the child to the question (child at 17 y old),”In the past 12 months, have you had a dry cough at night, apart from a cough associated with a cold or chest infection?” |
| Ever animal allergy | A positive answer from the caregiver to the question (child at 17y old),"Has your child ever experience allergic reactions such as hives, itching eyes, sneezing/nose bleeding, coughing, etc. by touching or approaching animals or entering the room where they were?" |
| Current animal allergy | A positive answer from the caregiver to the question (child at 17y old),"In the past 12 months, Has your child experience allergic reactions such as hives, itching eyes, sneezing/nose bleeding, coughing, etc. by touching or approaching animals or entering the room where they were?" |
| Ever food hypersensitivity | A positive answer from the caregiver to the question (child at 17 y old),"Has your child ever had symptoms such as urticaria and anaphylaxis (strong allergic reaction) after eating certain foods and been diagnosed with at food allergy by your doctor?" |
| Any allergy sensitization  (IgE component sensitization) | An Specific IgE ≥ 0.3 ISU for either component was considered positive.Serum levels of total and specific IgE measured using ImmunoCAP ISAC were collected. Specific IgE antibody levels were measured within the range of 0.3-100 ISU-E (ISAC standardized units). |
| Current pet ownership | A positive answer from the caregiver to the question (child at 13 y old),"Have you had a pet with hair (a dog, cat, bird, hamster, rabbit, etc.) at home during the last 12 months?" |
| Latex allergy | A positive answer from the caregiver to the question (child at 17y old),” Does your child get hives from touching rubber ?“ |
| Oral allergy syndrome | A positive answer from the child to the question (child at 17 y old),"Have you experienced itching in your mouth or throat or any other symptoms((Redness of the body and limbs, hives, coughing, wheezing, vomiting, diarrhea, abdominal pain, loss of consciousness, weakness, drop in blood pressure, etc.) throughout your body after eating raw fruits, vegetables　(cut fruit, salad, fresh juice, etc.), soybeans, processed soybean foods (Edamame, soy milk, etc.), or nuts?" However, children who answered only about systemic symptoms were excluded. |
| Sensitization of trees, grass, or weeds | Positive for any tree, grass, or weed component in ImmunoCAP ISAC. Included in ImmunoCAP ISAC are trees (Japanese cedar, Cypress, European white birch, alder,London plane tree, hazel, olive), grass, or weeds (Timothy, Bermuda grass, Lamb’squarters, English plantain, Annual mercury, Mugwort, Short ragweed, Pellitory of the wall, Saltwort). |
| Current pollen allergy | "Current Rhinitis at 17y" and "sensitization of trees, grass, or weeds" |
| PFAS | "Current Pollen allergy at 17y" and "OAS at 17y" |
